# 7 Dimensions of software change patterns

**DOI:** 10.1038/s41598-024-54894-0

**Published:** 2024-03-13

**Authors:** Mario Janke, Patrick Mäder

**Affiliations:** Data-intensive Systems and Visualization Group, Technische Universität Ilmenau, Helmholtzplatz, 98693 Ilmenau, Thüringen Germany

**Keywords:** Code changes, Data mining, Frequent graph mining, Auto-completion, Computer science, Scientific data, Software

## Abstract

Evolving software is a highly complex and creative problem in which a number of different strategies are used to solve the tasks at hand. These strategies and reoccurring coding patterns can offer insights into the process. However, they can be highly project or even task-specific. We aim to identify code change patterns in order to draw conclusions about the software development process. For this, we propose a novel way to calculate high-level file overarching diffs, and a novel way to parallelize pattern mining. In a study of 1000 Java projects, we mined and analyzed a total of 45,000 patterns. We present 13 patterns, showing extreme points of the 7 pattern categories we identified. We found that a large number of high-level change patterns exist and occur frequently. The majority of mined patterns were associated with a specific project and contributor, where and by whom it was more likely to be used. While a large number of different code change patterns are used, only a few, mostly unsurprising ones, are common under all circumstances. The majority of code change patterns are highly specific to different context factors that we further explore.

## Introduction

Software systems that are in active use are also under constant pressure to adapt and improve^1,2^. Maintaining software is a complex task that takes up to $70\%$ of overall software development effort^3^. Accurately predicting the effort of an ongoing project is an active research area on its own^4,5^. In order to keep software usable, source code has to be changed and expanded regularly in a non-trivial way. However, at many points, the same challenges arise over and over and thereby the resulting changes were shown to repeat across projects^6,7^. Patterns established themselves in form of the Gang of Four patterns^8^ as a tool to solve reoccurring challenges in a way that improves reusability, flexibility, and understandability^9^. Many of these patterns are still in very active use, such as Singleton, Factory, Adapter, Facade, Template Method, and Visitor. They also have remained subject of active and recent research. However, finding and applying the correct patterns needs time and expertise. In order to aid developers in this, numerous techniques were proposed, and the resulting patterns are widely applicable, e.g. for pattern-based autocompletion support in IDEs, modeling tools and the alike^10–12^. These techniques are based on project information, e.g. in the form of a natural text description^13^, a sequence of questions^14^ or a combination of stated user intent, class name relations, and others^15^. On the other hand, there are challenges that are entirely unique based on the unique nature of every software project and in fact, a number of change patterns are shown to be project-specific^16^.

When looking at the dynamic evolution of systems, patterns can be found in the way source code evolves, establishing the concept of *code change patterns*. These describe frequently performed procedures when altering code. From a software evolution standpoint, they allow drawing conclusions about reoccurring trends. Recent work applied different types of graph-based pattern mining to software evolution, allowing for an unbiased analysis^17,18^. This also opens up the possibility of analyzing more specific and niche patterns that are only relevant within a specific context.

In previous research, code change patterns have been analyzed based on their frequency measures^17–21^. By definition, a pattern must be reoccurring, which implies a certain necessary frequency. This makes frequency an important measure to judge a pattern.

However, the frequency always depends on the dataset that was used to calculate it. Consequently, the meaning of a *frequent pattern* changes based on the surrounding factors. A pattern that is frequent within a project could be a team-wide convention, while a pattern used by a single person could be a personal convention or habit. The goal of this work is to go beyond the frequency measure and to reflect *why* a pattern is frequent and within which surroundings, in order to make contextually relevant patterns easier to extract, and patterns easier to interpret. This is formulated in the following research question: **RQ1**: How do different factors of a software development process influence pattern occurrence?

To answer this RQ, different factors are identified and their statistical distributions are analyzed. Further, patterns that are at extreme points of the distributions are interpreted in a case study. From these results, we can aim to answer the question of whether these factors can be used as complementary measures to the frequency metric:

**RQ2**: Do the additional factors offer information beyond the frequency metric?

To answer this RQ, we analyze the correlation between different potential metrics and the frequency of a pattern. This also allows us to judge which measures should be seen in the context of the frequency, and which ones are suitable to use independently. Further, the patterns found by our stochastic mining method need to be reproducible in order to be valid. Thus we pose the third research question:

**RQ3**: Does the proposed method produce reproducible results despite its inherent randomness?

We aim to answer this RQ with an experiment in which the mining process is repeated 5 times independently. The results of all mining runs are then compared, to measure the reproducibility of the mining.

The main contributions of this paper are:

1. A novel approach for mining high-level file-overarching change patterns. Our approach can be used to find previously unknown patterns between different files as well as inside of files.
2. A large-scale study of 1000 open-source projects, producing a dataset of 45, 000 high-level change patterns, allowing for future mined patterns to be compared against the corpus.
3. A novel set of metrics to interpret and categorize patterns, classifying them along 7 different dimensions and comparing them to a large pattern baseline.

## Acquiring code patterns

**Figure 1:**
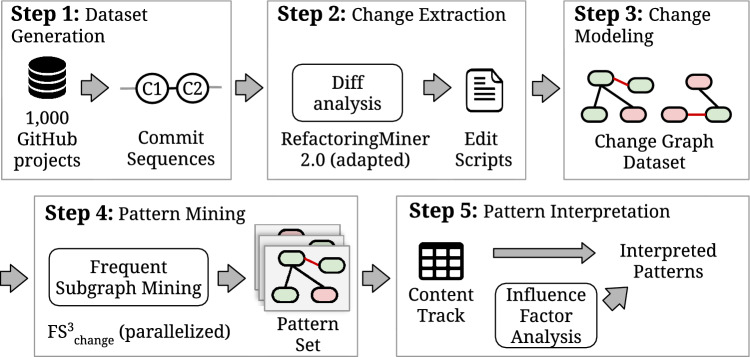
From GitHub projects to interpreted patterns - 5-step pattern acquisition process.

Previous code change pattern mining approaches were focused on small-scale low-level patterns. These offer insights into how the specific code structures change, but inherently lack the capacity to analyze high-level file-overarching changes such as a large number of refactorings. When analyzing change actions such as the extraction or splitting of a class, or an organized renaming of a class together with an interface, they cannot be connected and are interpreted as individual additions and deletions. This can lead to artifacts within the changes and misses the broader meaning of the change. In order to extract patterns, we build upon our FS^3^_change_ pattern mining process^21^ and adapt it for high-level file-overarching changes. Patterns are acquired in 5 steps that are presented in Fig. 1. Each step is detailed in the remainder of this section.

*Step 1: Dataset generation*

To build a corpus of software projects, we filtered for Java repositories on GitHub with a size of $>100$MB to ensure that the selected projects were well populated. This guarantees that the projects are predominantly Java-based, but may contain other types of code. A total of 92,588 projects passed the selection criteria. We then used stars as a metric to judge repositories’ popularity^22^ to select the top 1000 projects. The combined total of analyzed commits is 3, 989, 111. These changes were done by a total of 55, 007 distinct contributors.

*Step 2: Change extraction*

The most straightforward solution to extract changes between two code states is **text-based diffing**, which compares them line-wise. However, source code already has an underlying tree structure that is defined by how statements are nested inside each other, called the Abstract Syntax Tree (AST). While text-based diffing is very computationally efficient, using the AST allows us to represent the relations between different code elements.

For low-level code changes, GumTree^23^ can be used to diff individual files into a change representation called an edit script. An edit script is a sequence of edits, where each edit describes a change to a code token. An edit includes information about the type of change (addition, deletion, modification), the token’s type (class, operation, attribute, etc.), content (class name, etc.), and its position within the AST. It can include a reference to a parent edit if the token’s parent was also changed in the commit.

However, GumTree cannot be used to analyze multiple files in the context of the same commit at once. We overcame this restriction by adapting the source code of the open-source Java tool **RefactoringMiner** $\mathbf {2.0}$^24^ to produce commit-wise edit scripts. RefactoringMiner 2.0 detects a set of known refactorings within a code base. Since our goal is to search the data for previously unknown patterns, we still have to bridge a gap. To detect refactorings, RefactoringMiner builds a file-overarching representation of each commit and maps it to its respective following commit. These representations are then mapped to each other, similarly to the mapping step employed by GumTree. We reinterpret the code state representation of RefactoringMiner to generate an edit script-like syntax. This ultimately allows mining new patterns on the same bases previously known ones could be detected with, using the same methods that operate on edit scripts.

The resulting edit scripts are similarly structured to the ones produced by GumTree^23^ but leave out the lower-level diffs and cover the whole commit at once. They represent changes to classes, interfaces, methods, and attributes, but abstract from the individual lines of code and concrete implementations within the methods. Therefore, they have an about factor 3 lower number of edits compared to edit scripts generated by GumTree. For example, using GumTree, the addition of a variable is encoded in 3 tokens: *Add SingleVariableDeclaration*, *Add PrimitiveType* and *Add SimpleName*, while our method encodes this as a single token, *Add Attribute*. Using this method, we analyze all commits that both add and delete code tokens. Patterns within the low-level code structures have been analyzed in the past, leading to file-specific change patterns^17–19^. Looking at higher-level changes allows for finding different kinds of patterns that are focused on structure instead of implementation.

*Step 3: Change modeling*

From each edit script generated in step 2, a graph representation is built. The graph representation models the edits as labeled nodes, with labeled edges specifying how they are related. The specific types of tokens modeled by nodes are attributes, methods, classes, and interfaces. Additionally, generalizations describing inheritance between classes, and realizations describing when an interface is implemented, are added to the pool of nodes, in order to allow more complex relations between them. This also allows for keeping the important directness of these relations, even when working with undirected edges.

Based on the parent edit, *hierarchy* edges are added between nodes and their respective parent node. Based on the token content, a number of different edge types are calculated:

- sameContent: Describes when two tokens have identical contents. It can be used to detect multiple occurrences of the same method, multiple generalizations of the same class, etc.
- prefix: Describes when the content of a token starts with the content of another token, this can be used to detect relations such as between “logger” and “loggerFactory”.
- suffix: Describes when the content of a token ends with the content of another token, this can be used to detect related names such as “getValue” and “Value”.
- These relations are also analyzed with a case-insensitive variant, resulting in three additional edge types. This allows for modeling relations such as a variable named “logger” and a class “Logger” or those between “getValue” and “value”.

Each edit script representing one commit was transformed into a graph representation. The graph dataset consists of 7, 995, 753 nodes and 44, 445, 742 edges.

*Step 4: Pattern mining*

Every graph that appears frequently as a subgraph within the dataset’s graphs represents a frequent code change, i.e., a code change pattern. We chose FS^3^_change_ as the frequent subgraph mining algorithm. It is a stochastic FSM algorithm that repeatedly samples graphs from the dataset and stores candidates in a queue. Parameters are pattern size, number of samples, and the maximum number of output patterns. Regarding pattern size, a balance had to be chosen between occurrence frequency and pattern specificity. A small pattern is usually highly frequent but unspecific, while a large pattern is highly specific but not frequent. Pattern frequency tends to drop exponentially with pattern size^6^. In previous work^18^, patterns of sizes around 16 nodes were analyzed, showing frequencies around $1\%$. Since our pattern representation is more abstracted, and thereby multiple times more compact, we oriented towards the pattern frequency. We chose a number of 6 high-level edits to keep a sufficiently high number of complex interactions, and still find patterns with a frequency higher than $1\%$.

The original FS^3^_change_ approach joined the sets of change graphs from different software projects in advance of executing the frequent subgraph mining algorithm. This resulted in a huge memory requirement for this study, demanding over 200*GB* of RAM. We solved this by delaying the accumulation until after the pattern mining, thereby splitting the different processes. This effectively removes the memory bottleneck and allows for parallel execution of the frequent subgraph mining. We performed a separate frequent subgraph mining for each of the 1000 projects, with a maximum pattern number of 10, 000. It was done with 10 threads in parallel. The memory usage for this setup was 2*GB* per thread, which totaled 20*GB*. We then accumulated the patterns, which instantly gives an insight into the number of projects a pattern was found in. This resulted in a total of 2, 266, 371 distinct patterns. In order to increase confidence in the remaining patterns, we dropped all that were sampled less than 3 times, reducing the number of pattern candidates to 327, 785.

*Step 5: Pattern interpretation* Before the in-depth analysis of patterns, the numbers are reduced by removing the patterns with the lowest number of samples, as these are very likely to have a very low frequency. They are also harder to reproduce with subsequent runs of the same algorithm. Therefore, the most frequent 45, 000 patterns were selected as the base for an exact instance mining, in which the exact pattern frequencies and contents are acquired.

One of the key advantages of a large pattern corpus is that it can be queried through different lenses to find a set of patterns that is easier to oversee. A second key advantage is that any pattern can be compared against the corpus in different metrics, to see where it stands out. The instance mining returns the exact dataset-wide frequency of each pattern, the complete content track, as well as a detailed statistic of the context in which the patterns appeared. In the reproduction package (The dataset is available at: https://dataverse.harvard.edu/privateurl.xhtml?token=ad7488fa-6f47-4d36-866b-9b0a26a1077b) , the patterns are identified by their id. For each pattern, a full list of all occurrences is given, leading with the project id and instance id and then followed by the contents for each node. Additionally, the respective commit hashes are listed.

The gathered content and context distributions can now be analyzed using statistical methods. Further, this data can be reviewed for single patterns. Additionally, looking at commits containing instances of the pattern can be helpful for interpretation.

## Pattern influence factors

By applying the method to the dataset, we obtain a set of 45,000 patterns. These are referred to as P0 to P44999 based on the number of times they were sampled in the mining step. A pattern is characterized by its graph and the occurrences that can be interpreted using the content track. The meaning of a pattern is not solely defined by the graph, but also by all other constraints that can be inferred from the contents of each occurrence and how they relate. Typically, the graph structure gives a broad interpretation, while the instances fill in more specific details.

In order to answer RQ1 and RQ2, different influence factors are extracted and their impact on a pattern is analyzed. For RQ1, the distributions of these influence factors over all mined patterns are analyzed. Further, patterns that are extreme points of the resulting distributions are analyzed in detail, giving an understanding of the factors’ impact.

For RQ2, correlations between influence factors are calculated, uncovering whether they offer information over each other and how they relate to each other. To answer RQ3, the reproducibility is analyzed in a separate experiment, where the whole mining process is repeated 5 times. There is a great manifold of possible factors impacting software patterns - therefore it was necessary to do a selection based both on relevancy and availability within software repositories. Kelly et al.^25^ identified the software architecture and associated individuals as major impacting factors. We additionally reference factors such as the timeframe and the pattern frequency as pattern-specific metrics:

1. The number of **projects** the pattern occurs in, which differentiates team-specific or domain-specific patterns,
2. the number of **contributors**, which differentiates person-specific tasks,
3. the **edit types** of the edits that make the pattern, which differentiates refactoring patterns from code additions and deletions,
4. the **node and edge labels** of the pattern graph, which differentiates on which code elements the patterns operate and how they are related,
5. the **timing** of the pattern, which differentiates quickly reoccurring patterns,
6. the **context** of the pattern, which differentiates the surrounding in which a pattern happens, and
7. the **frequency** of the pattern, which differentiates how commonly used it is.

Along these dimensions, all patterns can be ranked, profiled, and classified. This allows viewing different categories of patterns, such as project-specific, widespread, addition, or refactoring patterns.

**Table 1:** Number of projects a pattern occurred in, average frequency per project, and percentage of occurrences that happen in the most contributing project of the pattern, with the respective minimum, 1st decile, average, median, 9th decile, maximum, and combined total over all commits.

	Min	${\text {D}_1}$	Avg	Median	${\text {D}_9}$	Max	All
Number of projects	1.0	97	212	200	346	684	1000
Avg. frequency per project	1.0	1.5	2.5	2.1	3.7	35.0	3997.1
Most contributing project	1.1%	2.0%	4.5%	3.4%	7.0%	100.0%	2.5%

### Project dimension

Software projects differ in structure, overall goal, and conventions. This can impact the way in which the source code evolves. Table 1 shows key values for this dimension. The ’number of projects’ row shows in how many projects a pattern occurred in. The values for the ’most contributing project’ describe the portion of pattern occurrences coming from the project with the highest number of occurrences of this pattern. Each pattern can have a different most contributing project, and the presented numbers are accumulated over them. A high number means a single project contributed strongly. The ’average frequency per project’ shows how often a pattern occurred within the same project again at a later point. For each row, the minimum, 1st decile, average, median, 9th decile, and maximum values over all patterns are shown. **All** represents the combined total over all commits, it is the equivalent of an empty pattern that occurs everywhere.

The average pattern occurs in $21.22\%$ of projects. The distribution of occurrences is uneven between projects, with the most contributing project accounting for about $4.5\%$ on average. In contrast to this, an even distribution between the average number of 212 projects would be $1/212 = 0.47\%$ proportion per project. This makes a pattern occurrence within the most contributing project about 10 times more likely on average.

**The average pattern analyzed occurred** $\mathbf {2.5}$ **times per project**. The 100 most frequent patterns occur 10.75 times per project on average. This, however, varies strongly between individual patterns with the existence of 9 patterns that are completely project-specific with all their occurrences being in the same project, and on the other hand, patterns that do not favor a specific project.

**Figure 2:**
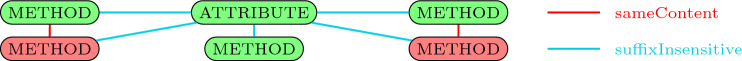
Pattern P289: Add attribute, update methods.

The pattern that is most evenly distributed among different projects is **Add attribute, update methods** (P289) (see Fig. 2, green highlighted nodes are *adds*, red nodes are *deletes*).

The graph is centered around a single added attribute that is suffix to 3 added methods. *Two* of them also have a deleted counterpart. We found 1, 703 different attributes, the most common of them being *name* ($4.2\%$), *id* ($3.9\%$), *value* ($2.2\%$), *type* ($2.2\%$) and *key* ($0.9\%$), with the methods typically being getters and setters. The method changes can happen in multiple ways such as movement between files and signature changes. This pattern is also a very general one, it shows that the introduction of an attribute *foo* likely impacts *getFoo*, *setFoo*, *createFoo* and *printFoo* type methods even if they are spread across different files.

**Figure 3:**
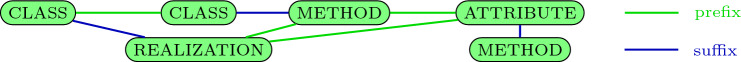
Pattern P24380: Add handler and reaction.

The opposing pattern is the most project-specific one, **Add handler and reaction** (P24380) (see Fig. 3).

At the center is a realization of an interface by a class that is also newly added. A related class is added and has a constructor added. Further, there is a variable named as a prefix that also has a suffix relation to another method. The pattern occurred exclusively in the project *rstudio*. It has unique contents every time. The central relation and the related class show to always end with “.Handler” while the other class and the related method end with “Event”. The single method always starts with “on”.

The pattern represents a project specific naming convention to implement an event handler and react to it. Within the single project it is used in a number of different contexts, never repeating a single variable name.

**Figure 4:**
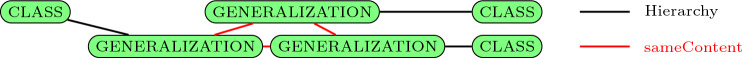
Pattern P5: Add 3 classes extending the same class.

Another notable pattern for this dimension is **Add** $\textbf{3}$ **classes extending the same class** (P5), as it occurs in the most projects (684) (see Fig. 4). As such, it is expected to be a very broad, very general pattern, that has no project-specific features in it.

There are 3 classes added which extend the same class. The extended classes are quite diverse with 5, 392 different names appearing, the most common extended classes are *TestCase* ($1.3\%$), *AbstractAction* ($1.1\%$), *CardImpl* ($0.9\%$) and *HttpServlet* ($0.6\%$). The extending classes are expectedly even more diverse, having up to 8, 748 unique contents, most commonly: *UnreferencedDatagramSockets.NamedWeak* ($0.1\%$), *edu.stanford.nlp.parser.shiftreduce.BinaryTransitionTest* ($0.1\%$) and *AESPBEWrapper* ($0.1\%$). Based on an inspection of instances, the pattern is often related to testing. An interface is often times extended multiple times within the same commit. This seems to be a very project-overarching working pattern.

**Table 2:** Number of contributors, the average number of contributions per person, and percentage of occurrences by the most contributing contributor of the pattern.

	Min	${\text {D}_1}$	Avg	Median	${\text {D}_9}$	Max	All
Number of contributors	1	120	394	309	765	4219	55,007
Avg. freq. per contribution	1.0	1.2	1.4	1.4	1.7	38.0	72.5
Most contributing cont.	0.5%	1.1%	3.1%	2.1%	5.1%	100.0%	0.5%

### Contributor dimension

Contributors differ in preferred tasks, experience and coding style, which can influence their coding structure. Table 2 shows the number of contributors, the highest relative portion added to the pattern occurrences by a single contributor, and the average number of pattern occurrences per person. The average pattern is shared between 394 out of 55, 007 contributors which makes for $0.72\%$ of contributors. The most contributing contributor on average committed $3.07\%$ of occurrences of a pattern, an even split would have been $0.25\%$. This makes the most contributing contributor more than 12 times more relevant than on average. This hints at the possibility of change patterns having an ownership dynamic similar to static code patterns. Bird et al.^26^ noticed a large portion of over $40\%$ of commits coming from a single top contributor. They also observed an increased number of faults resulting from contributors with fewer commits.

**The average pattern was used** $\mathbf {1.5}$ **times by each contributor, a top-**$\textbf{100}$ **pattern was used** $\mathbf {2.2}$ **times**.

The number of contributors varies strongly between patterns. There are 3 patterns that are only used by a single person. The most contributors sharing a pattern were 4, 219. The number of contributors is closely connected with the number of projects a pattern occurred in. Out of the $10\%$ (4, 500) of patterns with the highest contributor count, 3, 967 were also within the $10\%$ contributing to the most projects.

**Figure 5:**
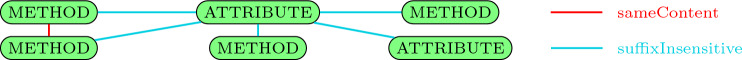
Pattern P47: Propagated signature change towards method invocations.

The most general pattern between contributors is **Propagated signature change towards method invocations** (P47) (see Fig. 5).

A single added attribute is connected to 4 added methods and another attribute. The name of the attribute is a suffix to all of them. Two methods are identically named. The most common attributes are *id* ($8.1\%$), *name* ($5.7\%$) and *type* ($3.1\%$). The most common methods are respective *get* and *set*, *is* and *set*. The secondary attributes are more specific versions of the first one like *serialVersionUID* ($2.0\%$), *friendlyType* ($0.4\%$) or *userId* ($0.2\%$). The pattern is quite frequent with 6, 819 occurrences and 2, 989 contributors across 627 projects. The contributor with the highest contribution to this pattern only accounted for $0.5\%$ of the occurrences. The attribute often is added in multiple files within the same commit. The pattern is unlikely to be used excessively by any single developer but likely to be used once.

**Figure 6:**
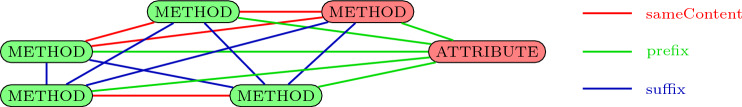
Pattern P22211: Refactoring around graph *g*.

The most contributor-specific pattern, **Refactoring around graph** *g* (P22211), was used only by a single developer in two projects. It describes a refactoring around a graph *g* and its related variables starting with *g* (see Fig. 6).

The graph consists of three connected groups with identical contents of size 3, 2 and 1. They consist of 2 method additions and a deletion; 2 method additions and an attribute deletion, respectively. The size 2 group is in a suffix relation to the size 3 one, both of them have the single node as prefix. The larger group of methods reveal to be *g_v1_out_out_treeXnameX* ($5.3\%$), *g_v1_out_sideEffectXincr_cX_valueXnameX* ($5.3\%$), *g_V_outXcreatedX_groupCountXnameX* ($5.3\%$) while the smaller ones are related getters. The single node always is the letter *g*, which showed to be a personal convention for the variable name of type *graph*. The deletion can be caused by removal of a whole file. The pattern describes a series of refactorings done by a single person, adapting it to a new model. It presents itself as a pattern because of the strict naming conventions used.

**Table 3:** Distribution of different edit types within the patterns $\textbf{f}_{{\textbf {pattern}}}$, percentage of patterns with at least one edit of specific type $\textbf{f}_{{\textbf {pattern}}}^{\ge 1}$, in the data $\mathbf {f_{data}}$ and in transactions with at least a single occurrence normalized to 1, $\textbf{f}_{{\textbf {data}}}^{\ge 1}$.

Edit type	$\text {f}_{\text {pattern}}[\%]$	$\text {f}_{\text {pattern}}^{\ge 1}[\%]$	$\text {f}_{\text {data}}[\%]$	$\text {f}_{\text {data}}^{\ge 1}[\%]$
Add	73.41	88.78	71.52	69.49
Delete	25.20	43.95	26.51	28.62
Move	1.39	4.13	1.97	1.89

Change patterns that occur between two or more files are a strong hint at dependencies between these files. Lambers et al.^27^ analyze conflicts and dependencies using graph transformation. Pruijit et al.^28^ examined 10 architecture compliance checking tools on a custom Java corpus, reporting an accuracy of around 75%. Change patterns offer another alternate lens on how code pieces can be related by uncovering code that is edited together rather than having a direct code-level dependency. As examined by^26^, there is a relation between code that has a dependency and code that is worked on by the same contributors.

### Edit dimension

Code edits can be pure additions or deletions, but also a mixture of different types of actions. Table 3 shows the frequency of edit types in different contexts. The column $\textbf{f}_{{\textbf {pattern}}}$ is the distribution within a pattern. $\textbf{f}_{{\textbf {pattern}}}^{\ge 1}$ shows the proportion of patterns with at least one of the edit types in them. For this measure, a pattern can be counted in multiple categories at once. $\textbf{f}_{{\textbf {data}}}$ is the distribution within the whole dataset and $\textbf{f}_{{\textbf {data}}}^{\ge 1}$ is the proportion of graphs with at least one node of the respective edit type in them. The most present edit type is *add*, which is about 3 times more likely than *delete*. *mov* is only rarely detected with almost $2\%$. When looking at the patterns, *add* also is dominant. This is to be expected, as frequent patterns are likely to be built from frequent nodes. However, it is remarkable that *del* is only about $1.3\%$ less frequent in the patterns than in the whole dataset. Looking at the $\textbf{f}_{{\textbf {pattern}}}^{\ge 1}$-column, we can see that $>11\%$ of patterns do not have an *add* in them. Also, there are still $1.4\%$ of *mov* nodes within the patterns. This means that there are **many frequent patterns using these infrequent node types.** The number of patterns with both an *add* and *del* in them is $32.73\%$. This is remarkably high, as there is a high likelihood of these patterns representing a refactoring of some kind. Of the patterns, $54\%$ had only *add*s and $11\%$ only *del*s. The most frequent pattern (P0) mined has *add*s and *del*s (see “Pattern frequency dimension” section).

**Figure 7:**
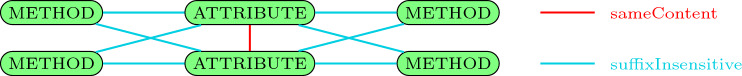
Pattern P27: Add two attributes and methods to interact with them.

The most frequent pattern that consists solely of additions is **Add two attributes and methods to interact with them** (P27) (see Fig. 7).

The pattern is centered around 2 additions of identically named attributes. They are suffix-related with 4 added methods. The most common attributes with this pattern are *id* ($4.2\%$), *name* ($3.5\%$) and *value* ($1.6\%$). Besides *get* and *set*, there are other ways of interaction like *convert*, *create*, *findBy*, or *subscribe*. The attributes are added within multiple different files. It is a very common naming scheme to name methods based on the variables they interact with. Such methods are commonly added within the same commit as the variables. It could be seen as a warning sign when such a method does not interact with the variable.

**Figure 8:**
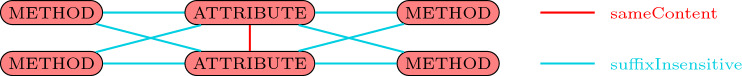
Pattern P453: Delete two attributes and methods to interact with them.

The most frequent deletion pattern is **Delete two attributes and methods to interact with them** (P453), it describes the deletion of the structure added in the pattern P27 discussed above (see Fig. 8). The structure is identical to P27 apart from all node types being deletes. The most common attributes are *name* ($4.2\%$), *id* ($4.1\%$), *type* ($1.6\%$), with the methods typically being *get*, *set* or *is*. Typically, in some form of code restructuring, a variable gets removed together with all methods specially made to interact with it. This pattern is complementary to P27, as it seems to remove exactly what was added. There are some slight variations in the frequencies, but the overall variables and methods are very similar. It is $38\%$ as frequent as P27 which gives an estimation of how often such a feature is removed later on.

**Table 4:** Distribution of the token type frequency within the dataset $\textbf{f}_{{\textbf {data}}}$, within the patterns $\textbf{f}_{{\textbf {pattern}}}$ and the percentage of patterns with at least one occurrence of the specific token type $\textbf{f}_{{\textbf {pattern}}}^{\ge 1}$.

Token type	$\text {f}_{\text {data}}[\%]$	$\text {f}_{\text {pattern}}[\%]$	$\text {f}_{\text {pattern}}^{\ge 1}[\%]$
Class	10.75	20.5	60.12
Method	61.15	39.8	80.84
Attribute	13.94	20.8	58.80
Interface	0.67	0.3	1.34
Generalization	8.77	10.2	31.13
Realization	4.72	8.3	27.80

**Table 5:** Distribution of edge types in the data $\textbf{f}_{{\textbf {data}}}$, within transactions with at least one edge of specific type $\textbf{f}_{{\textbf {data}}}^{\ge 1}$, in the patterns $\textbf{f}_{{\textbf {pattern}}}$ and the percentage of patterns with at least one occurrence $\textbf{f}_{{\textbf {pattern}}}^{\ge 1}$.

Edge type	$\text {f}_{\text {data}}[\%]$	$\text {f}_{\text {data}}^{\ge 1}[\%]$	$\text {f}_{\text {pattern}}[\%]$	$\text {f}_{\text {pattern}}^{\ge 1}[\%]$
Hierarchy	0.95	8.74	6.0	25.78
SameContent	62.05	38.84	25.4	66.34
Prefix	14.66	17.11	25.7	58.78
SameContentI	0.72	2.35	1.3	4.50
PrefixI	2.39	5.14	6.7	18.87
Suffix	9.96	13.94	33.0	55.35
SuffixI	9.26	13.87	35.7	55.13

### Structural dimension

Patterns can be differentiated along the types of code tokens they engage with as well as their respective contextual connections. Table 4 shows the distribution of object types for the dataset and within patterns. The frequency $\textbf{f}_{{\textbf {data}}}$ describes how frequent the object type is relative to the other ones within the graph dataset. $\textbf{f}_{{\textbf {pattern}}}$ is a similar measure but within the patterns. $\textbf{f}_{{\textbf {pattern}}}^{\ge 1}$ is the fraction of patterns with at least one object of this type , where a pattern can be part of multiple categories. The most frequent type in the data and in patterns is *method*, however, its frequency is relatively lower in patterns. The difference in frequency describes how pattern-affine an object type is. The strongest gains can be seen for classes and attributes (from $11\%$ / $14\%$ to $21\%$ / $21\%$). About $60\%$ of patterns had at least one class in them, and $59\%$ had an attribute. Based on this, **classes and, to a lesser extent, attributes seem to play a central role in change patterns**.

Table 5 shows the frequency of individual edge types. $\textbf{f}_{{\textbf {data}}}$ describes edge type frequencies in the data. $\textbf{f}_{{\textbf {pattern}}}$ describes edge type frequencies in the patterns. $\textbf{f}_{{\textbf {pattern}}}^{\ge 1}$ describes edge type frequencies with at least one occurrence of the respective edge type, where a pattern can be part of multiple categories. $\textbf{f}_{{\textbf {data}}}^{\ge 1}[\%]$ describes the proportion of graphs with at least a single edge of the specific type in it.

**Figure 9:**
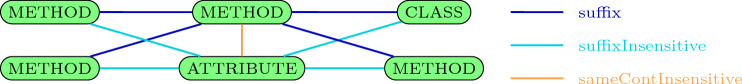
Pattern P1109: Add variable named like class declared with constructor.

The most frequent pattern with a same content insensitive edge is **Add variable named like class declared with constructor** (P1109) (see Fig. 9).

The pattern is structured around an attribute and a method that are named identically except for the case. Their names are suffix to 3 methods and a class. The most frequent attribute names are *criteria* ($1.1\%$), *message* ($0.9\%$), *type* ($0.9\%$) with the method being identical except starting with an upper case letter. The most common methods are *getOredCriteria* ($1.1\%$), *createCriteria* ($1.1\%$) and *GeneratedCriteria* ($1.1\%$). The core method is the constructor of the related class. Naming a variable similar to a type is a quite common naming scheme, especially when there only is a single instance of that type in the scope. These kinds of variables are very often introduced in the same commit as the types and are more likely to use the constructor of the type.

**Figure 10:**
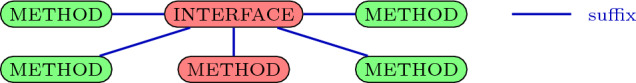
Pattern P12121: Replace interface with methods.

The most frequent pattern using an *interface* is **Replace interface with methods** (P12121) (see Fig. 10).

The pattern is structured around the deletion of an interface. 4 methods are added that have an suffix relation to the interface and 1 is deleted. The interfaces and methods are highly varied, the most common interfacs are: *Iterator* ($3.5\%$), *Filter* ($1.5\%$) and *DelegatableDecoder* ($1.3\%$). The methods tend to interact with the interface by verbs like *get*, *close* or *tryGet*. The functionality of an interface is replaced by some different code construct. In some cases this can be as simple as renaming it, but typically its functionality is absorbed by methods or an already existing interface.

**Table 6:** Time difference between occurrences of the same pattern (**Diff**) and expected time diff. based on pattern frequency (**E(Diff**)).

	Min	${\text {D}_1}$	Avg	Median	${\text {D}_9}$	Max	All
Diff	2.1	7862.1	11,932.9	11,762.0	16,238.9	441,461.7	14.0
E(Diff)	4097.3	45,616.4	92,252.6	133575.4	364,931.5	2,938,658.9	14.0

### Timing dimension

The time between pattern occurrences differs between patterns based on their structure. Table 6 shows the time difference between commits a specific pattern was found in within the respective projects. Over the whole dataset, the average time between commits within the same project was 14*h*. The expected time describes how much of a time gap is likely based on the frequencies of the respective patterns. **The time span between two consecutive occurrences of the average pattern is** $\mathbf {7.7}$ **times shorter than expected**. **Out of the top** $\textbf{100}$ **patterns, one is used every** $\textbf{14}$ **hours on average.**

The patterns with the $10\%$ smallest gaps between occurrences have an average frequency 2.3 times higher than the overall average, while the number of projects they occur in is only 1.2 higher. This suggests that patterns used in rapid successions tend to be more project specific. The number of contributors is 1.8 times the average, but the most contributing contributor has 2.5 times more impact. This suggests that patterns used in rapid succession are often carried out by a low number of individuals.

**Figure 11:**
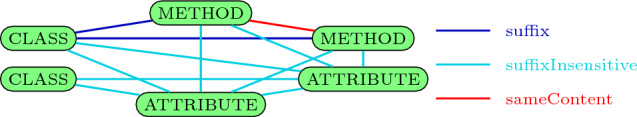
Pattern P44628: Suffix chains.

The pattern happening in most rapid succession (P22211) is also the one used by the least contributors (see “Contributor dimension” section). The one with the largest time gaps in between appearances are **Suffix chains** (P44628) (see Fig. 11). The pattern describes a chain of growing variable and class names.

The pattern consists of 2 attributes, 2 classes and 2 methods whose names relate by chaining different kind of suffixes. The content varies strongly, with almost no content repeating. One example is the factory pattern around the (constructor) method *ReflectionEnvironmentPostProcessorsFactory* with attributes *postProcessorsFactory* and *factory*, and the classes *ReflectionEnvironmentPostProcessorsFactory* and *EnvironmentPostProcessorsFactory*. It occurred 60 times in 55 different projects. The attribute name typically comes down to the naming preference of a variable being named like a type when only a single variable of that type is feasible in that context. The pattern shows the interplay of class hierarchy and this naming convention. It applies to a large variety of different contexts, however the factory pattern seems to be the most common usage.

**Table 7:** Pattern frequency, number of files changed, number of lines added, and number of lines deleted accumulated over pattern occurrences.

	Min	${\text {D}_1}$	Avg	Median	${\text {D}_9}$	Max	All
Frequency	19	153	606.4	418	1225	13,627	3,989,111
#Files changed	3.7	33.2	50.9	47.7	66.7	427.5	11.8
#Lines added	71.3	1109.9	2380.2	2244.3	3505.4	43,186.8	422.5
#Lines deleted	40.3	406.0	1219.8	878.9	2437.4	13,896.3	312.0

### Edit context dimension

The size and type of a commit can influence the patterns contained within it. Table 7 shows how the number of files changed, code lines added and code lines deleted accumulated over all commits in which the pattern occurred. Looking at the number of files touched, the average in the data is 11.8, while the average in the pattern is 50.9. Similarly, patterns tend to happen more in changes with more additions and deletions. This is likely because a larger change gives the possibility for more connections within the data. The number of changed files varies greatly over patterns, with the lowest one being 3.7 up to a maximum of 427.5. **The average number of files changed for a purely additive pattern is **$\textbf{43}$ **while it is** $\mathbf {59.7}$ **for pure deletions**.

**Figure 12:**
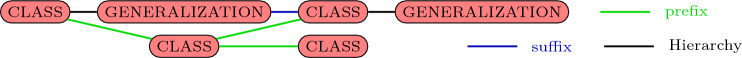
Pattern P34266: Remove class hierarchy.

The pattern resulting in the most changed files is **Remove class hierarchy** (P34266) (see Fig. 12).

The pattern is centered around the deletion of 4 related classes and 2 realizations. The class names vary widely, the most frequent ones being *EntryWithValue* ($1.9\%$), *Entry* ($1.9\%$), *ConcurrentWeakMap* ($1.9\%$) and *Segment* ($1.9\%$). The most frequently implemented interfaces are *ContextPrint* ($2.5\%$) and *RuntimeException* ($3.1\%$). It has the highest number of files changed in an average occurrence with 428. And a substantial number of lines deleted with 6, 227. The pattern is strongly correlated with large and delete-heavy commits. This likely is because a whole hierarchy of classes is removed at once.

### Pattern frequency dimension

A very frequent pattern is likely structured differently than a less frequent one. Patterns’ frequencies ranged from 19 to 13, 627. **The average pattern occurs** $\textbf{605}$ **times.** There is a small number of very frequent patterns that rapidly falls off. The sum of all patterns’ frequencies was 27, 289, 914, **resulting in about** $\mathbf {6.8}$ **occurrences per commit**. The average deletion pattern is $28\%$ less frequent than average.

**Figure 13:**
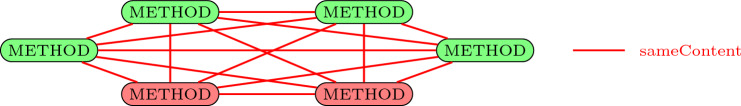
Pattern P0: Mass Change Method Signature.

The most frequent pattern is **Mass Change Method Signature** (P0) (see Fig. 13).

A number of 4 identically named methods are added. Additionally, 2 methods with the same name are deleted. There are 8, 596 different methods affected by this pattern, the most common ones being: *create* ($1.6\%$), *visit* ($1.5\%$), *execute* ($1.4\%$) and *get* ($1.1\%$). Usually, a method used very often within the commit receives impactful changes to its signature and/or modifiers. As this pattern is the most frequent, it is expected to be very generic. It represents an update to a method’s signature that is applied to many parts of the code.

**Figure 14:**
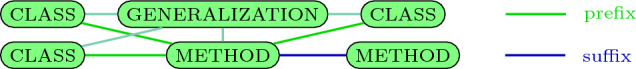
Pattern P43357: Add serializer and deserializer.

The least frequent pattern mined is **Add serializer and deserializer** (P43357) (see Fig. 14).

The pattern is centered around an added method. It has a prefix relation to 3 added classes and an added generalization. Also, there is another suffix-related added method. The central method is always *serialize* and the related one is *deserialize*. One of the classes is always a *Serializer*. The other classes never repeat and represent different subtypes of serializers such as *GenericSerializerWithObjectReuse*. The pattern was exclusive to the project *eishay/jvm-serializers*. This pattern represents a specific way of building and naming serializers and deserializers.

**Table 8:** Correlation coefficients of pattern frequency with other pattern metrics.

Metric	Correlation Factor
Number of contributors	0.96
Number of projects	0.81
Number of tokens with type ’Operation’	0.27
Number of edges with type ’Content Prefix’	0.18
Number of lines added in instance commits	0.06
Number of edits of type ’Add’	0.02
Number of lines deleted in instance commits	− 0.04
Number of files changed in instance commits	− 0.05
Number of tokens with type ’Class’	− 0.09
Average time between commits	− 0.46

Table 8 shows the correlation between frequency and metrics from the other dimensions. A correlation factor of 1 means that both rankings are always identical, $-1$ means that they are always exactly opposite, and 0 means that there is no correlation at all. There are very strong positive correlations, uncorrelated metrics, and negative correlations. By far the strongest correlation with the frequency is the number of contributors of a pattern, followed by the number of projects the pattern occurred in. There are weaker correlations with the number of *operation* nodes and *prefix* edges. There also is a negative correlation with the average time between commits. Measures that are strongly correlated with the frequency, such as the number of contributors or projects have a high overlap with the frequency. Interpretation using these measures should be aware of the correlations. For example, a pattern might be especially interesting because it only occurred in a small number of projects *despite* its high frequency. Another pattern might stand out because of a large number of contributors despite comparatively low frequency. This is also true for negatively correlated measures, since a negative correlation with a high frequency is a positive correlation with a low frequency. It can be seen that none of the factors are identical to the frequency metric, which means that they offer information beyond the frequency metric. This is discussed further in “RQ2: Do the additional factors offer information beyond the frequency metric?” section.

### Pattern profiles

In this section, 13 patterns that represent extreme points of the analyzed dimensions were interpreted. Table 9 shows the profiles of these patterns based on their ranking within the different dimensions, compared to all other mined patterns, as quantiles.

**Table 9:** Quantiles of 13 interpreted patterns for the average time in between occurrences, number of contributors, number of projects, the average number of changed files, the average number of lines added and deleted, and frequency.

Name	Time (%)	Contr. (%)	Projects (%)	Files (%)	Lines+ (%)	Lines- (%)	Frequency (%)
P5	0.7	99.9	**100.0**	15.3	67.8	47.2	99.9
P289	3.6	99.2	99.3	76.5	68.5	56.7	99.1
P24380	0.3	0.1	*0.0*	0.0	0.0	0.0	0.1
P47	1.1	99.9	99.9	28.8	57.0	36.4	99.8
P22211	0.0	*0.0*	0.0	0.0	0.0	0.0	0.1
P27	0.4	99.9	99.9	22.9	49.8	59.1	99.9
P453	2.2	99.4	99.8	63.4	9.2	81.4	99.5
P1109	20.1	94.4	95.4	18.1	67.7	49.7	92.6
P12121	48.3	68.2	62.1	70.0	46.4	59.3	72.0
P44628	**100.0**	0.9	1.5	40.5	76.3	69.0	0.6
P34266	73.5	13.1	12.1	**100.0**	83.9	99.5	10.8
P0	0.3	100.0	99.9	24.1	20.0	51.6	**100.0**
P43357	2.0	0.2	0.0	0.0	2.0	0.0	*0.0*

Dimension maxima are highlighted in bold, and minima in italic.

Pattern P5, for example, was selected because it occurred in the highest number of projects. Naturally, it also ranks high in regard to the number of contributors and the overall frequency. However, it is neither the most frequent one nor the one with the most contributors. Looking at the other dimensions, it can be seen that P5 occurred in commits with below-average numbers of edited files, which had an above-average overall number of added lines, meaning that it tends to occur within large changes.

Patterns can also be ranked in other properties not represented in the table, such as the impact of their most active contributor (3.2% for P5) and of their most relevant project (2.2%) as well as the graph structure. P5 shows to have a high number of classes (ranking top 81.5%), generalizations (91.16%), number of hierarchy edges (98.3%) and number of sameContent edges (77.3%). Ranking patterns along these dimensions can be used to interpret individual patterns, giving additional insights into the circumstances in which a pattern is used.

**Pattern applications.** It can be seen that the patterns that are the most general, such as P5 (used in most projects), P27 (most frequent additive pattern), and P0 (found most often overall) can be found in so many situations that they might lack the specificness necessary to be helpful in practice. Less frequent patterns, that are more situational, can also be more specific and stand out in other metrics. P24380, for example, describes a certain way in which events can be handled. When this pattern applies, the handler of an event could be automatically prepared after the interface has been realized.

P47 is an example of a more general pattern, it describes a method signature change that is propagated throughout the code base. While the pattern itself is unsurprising, it still shows potential to assist a developer in performing it. When the start of the pattern is detected, all relevant parts of the code base that need to be adapted could be highlighted, and the signature change could be offered for autocompletion.

### Reproducibility analysis

To evaluate the stability of the proposed method, we repeated the non-deterministic part of the mining pipeline 5 times. This allows us to compare the results and infer statements about the method’s reproducibility.

**Table 10:** Average and standard deviation for the number of patterns (**p**), average number of patterns that also occur in the top **p** patterns of reference runs (**p-p-cut**), number of patterns that occur in the reference run (**p-all-cut**), difference in samples between multiple runs (**sample diff**) and difference in project number estimated between multiple runs (**project diff**).

p	p-p-cut	p-all-cut	Sample diff	Project diff
1k	$974 \pm \,\ 1.9$	$1000 \pm 0.0$	$2.87\% \pm 0.04\%$	$0.24\% \pm 0.010\%$
10k	$9,392 \pm 11.2$	$10,000 \pm 0.0$	$9.54\% \pm 0.07\%$	$0.59\% \pm 0.003\%$
100k	$85,455 \pm 73.2$	$99,698 \pm 9.1$	$36.37\% \pm 0.19\%$	$4.45\% \pm 0.030\%$

Table 10 shows the stability measures for the first $p=$ 1*k*, 10*k* and 100*k* patterns. The $\textbf{p}$**-**$\textbf{p}$**-cut** shows how many of the top *p* patterns mined in the baseline run are also included in the top *p* patterns in the repeated runs, while the $\textbf{p}$**-all-cut** searches within all mined patterns. The **sample diff** describes the difference in samples between the respective runs, while the **project diff** shows the difference in the number of projects that contributed such a pattern. The sample inaccuracy depends on the pattern ranking, going from less than $3\%$ up to $36\%$. The variation in the number of projects contributing a sample is much lower, ranging from $0.2\%$ to $4.5\%$. Within the *p*-all-cut, all patterns occurred in all runs for 1*k* and 10*k*, while for 100*k*, a number of 302 did not occur on average. The *p*-*p*-cut is a much stricter measure, and finding all patterns in the top *p* of all other runs would mean they are fully identical every time. The repeated runs overlapped by $97\%$, $94\%$ and $85\%$ of patterns, for $p=1k$, $p=10k$ and $p=100k$, respectively. The quality of estimations generally falls with higher numbers of patterns. This is because they are sampled much less frequently. Still, even at 100*k* patterns analyzed, around $85\%$ of patterns were in the top 100*k* repeatedly, and the number of samples differed only by $36\%$.

## Discussion

### RQ1: How do different factors of a software development process influence pattern occurrence?

The 45, 000 analyzed patterns showed variations based on their distribution over projects and contributors, the edit types and code tokens used, their distribution over time and commit sizes as well as their frequency. The analysis resulted in the following findings:

- Projects have key patterns that they use frequently. These patterns vary a lot from project to project, and often are not very prominent in other projects.
- Contributors have individual, frequently reused patterns. These patterns vary between contributors. Some contributors use their patterns across multiple projects.
- A contributor is likely to use a pattern multiple times in a short timespan. On average, the same pattern occurred again 7.7 times earlier than expected.
- A large share of patterns is related to change, such as refactorings, fixes and maintenance. 1 in 3 patterns (14, 730 in total) consists of additions *and* deletions, meaning that it is focused on modification of behavior rather than pure addition. This is supported by 50% to 80% of software cost being related to maintenance activities^29^.
- More frequent patterns are structurally different from less frequent ones, on average, they are centered more around operations and prefix relations.
- While the addition of new code is typically incremental, deletions often happen in large file-overarching bulks. The focus of a large deletion lies in finding all occurrences across the whole codebase.

The analyzed patterns give insight into the way software structure is altered over a project’s lifetime. Patterns show to fall into one of two categories: (1) Widely adopted project-overarching changes. These are used by a high number of contributors. Thus, they cannot be specific to a project or an individual. They are much more concerned with refactorings, such as introducing a variable and adding it to signatures or replacing a barely used interface with merely a method. These refactorings tend to be more general, as they are not concerned with the actual software structure and instead more with their alteration.

The other category consists of (2) Personal and project-specific patterns. These are the ones with the highest potential to be used intensively by a specific developer. They are more focused on additions of new functionality, specifically involving classes. They are unlikely to be used outside of their specific context because they interact with a specific code constellation or use a specific naming scheme, for example. In the study by Nguyen et al.^17^, 43% shared all their patterns with other developers, and 88% shared at least 10% of their patterns. They argue that this means that these patterns are *pervasive among developers*. This is consistent with category (1) of patterns identified in our study. They also found that 46% of pattern instances are repeated by the same developer within a year. Our study confirmed that patterns are more likely to be repeated soon after their occurrence by the same developer.

*Answer*: Different factors can have a defining impact on the structure of a pattern. A pattern that is general over a high number and projects and contributors cannot contain specific code conventions or be focused on a certain domain. On the other hand, project-specific patterns can be very important for a team, and yet far below frequent on a general scale.

### RQ2: Do the additional factors offer information beyond the frequency metric?

The correlation factors in Table 8 showed that the different dimensions relate in various different ways to pattern frequency. The strongest correlations with the frequency were the number of projects the pattern occurred in and the number of contributors using it.

Looking at Table 9, it can be seen the pattern appearing in the most projects is different from the one with the highest frequency, but both are ranked very highly in the other categories. Interestingly, in the pattern inspection, both patterns showed to be very different, with P5 (most projects) being purely additive and focused on class hierarchy, while P0 (highest frequency) is a refactoring of methods. Still, because of the high correlation, it might be beneficial to calculate project/contributor-specificity relative to the frequency.

The measures regarding the context of the commit were mostly uncorrelated with the pattern frequency, while still having a rich distribution within them (see Table 7), making them an orthogonal way to look at a pattern. The time between commits that include the same pattern is negatively associated with the frequency. A pattern with more occurrences naturally has a smaller expected time until its next occurrence. Interestingly, the correlation is far weaker than the ones regarding the number of projects or contributors. This shows that the timing is not solely dependent on the frequency, and suggests that there inherently are patterns used sparingly and others used multiple times in conjunction even when controlling for frequency. There also is a week correlation towards the structure of a pattern, with a slight over-representation of operations and the prefix-edge type.

*Answer:* Yes, the dimensions offer additional information beyond the frequency measure. Two measures (contributors and projects) were highly correlated to frequency, and while still offering additional information, should be seen in context to the frequency.

### RQ3: Does the proposed method produce reproducible results despite its inherent randomness?

Looking at Table 10, the *p*-all-cut showed that the patterns found are very similar within the different runs. The *p*-*p*-cut showed that the pattern rankings do not differ by much. The variation in project number is remarkably low. This shows that patterns tend to come up within the same projects within different runs.

The frequent subgraph sampling results showed to be highly reproducible despite the randomized nature of the mining process. This can be explained by the very high number of samples drawn. The frequencies can even out within the 100, 000 samples and between the 1000 projects. However, there still is a variation within the number of samples drawn for each pattern candidate and therefore, within their rankings before the instance mining. The variations are generally very low, however, they get larger for patterns with lower sample numbers. This is because of less possibility to average out sampling variations. This also is an argument for the reliability of both FS^3^^30^ and FS^3^_change_ and shows that the memory bottleneck can be mitigated effectively.

*Answer:* Yes, the process could be shown to be highly reproducible, especially in regard to patterns that are sampled sufficiently often.

### Implications

#### General takeaways

General patterns that are important almost regardless of the specific situation do exist.

However, most patterns are bound to a specific context. On average, the contributor using a pattern the most uses it 10 times more than the average contributor. Similarly, many patterns are far more likely to be predominately used within a small set of projects. Within their context, these patterns are as important or even more important than general ones, despite them being harder to extract due to a lower frequency overall.

#### Takeaways for developers

Not every pattern suits every team or software. The unique challenges faced with every software project and team constellation are also reflected in the patterns used. A pattern that is generally highly relevant might still be unsuitable for the specific situation. On the other hand, it is a common occurrence for teams and individuals to have their own process.

#### Takeaways for IDE/language developers

Recommendations that are based on known patterns, such as autocompletion and refactorings could benefit from context awareness. For example, a refactoring that was used by the same team in the past, or a different team in a comparable situation could be recommended with a higher priority.

#### Takeaways for researchers

Understanding and interpreting a pattern is a difficult task. Frequency as a measure is valuable but limited, as it only considers a single pattern without comparison to others. Interesting and relevant patterns are often hidden behind general frequent ones. Pattern profiles offer more information for judging patterns and can help to rely less on the interpretation of specific pattern occurrences (as demonstrated in “Pattern profiles” section).

Apart from that, patterns can be ordered and queried based on desired properties, allowing for the extraction of the relevant ones. Past research has been mostly focusing on patterns that are general across different contexts. Context-specific patterns have the potential to complement that research and further improve tools such as autocompletion or refactoring recommendations by offering more specific suggestions.

## Limitations and threats to validity

### Limitations

The mining method has certain limitations. Both the subgraph mining and the pattern metrics proposed in this work are language-agnostic. However, the current iteration of the diffing method is based on RefactoringMiner 2.0, which is limited to Java code. A possible approach to support a multitude of programming languages could be to extend the functionality of language-agnostic diffing tool such as GumTree to cover file-overarching commits.

The patterns analyzed were high-level patterns. There are differences compared to low-level patterns such as those discussed in previous works^17,18^. Low-level patterns are concerned with the alternations of variables on a line level mostly contained within a single method. High-level patterns focus on changes in classes, interfaces, and method signatures as well as their movement between different files. Many parts of this work naturally generalize to low-level patterns:

- The classification of patterns into different dimensions,
- all proposed interpretation metrics,
- the optimizations applied to FS^3^_change_.

Apart from that, there are limitations in analyzing software repositories that use a version control system. Changes that are reverted within a commit are not represented in the repository and therefore, patterns within these actions cannot be mined. This becomes especially relevant with very large commits^31^.

### Internal validity

Even though results are shown to be mostly stable for the given dataset, the mining method is still non-deterministic and subject to smaller variations between different runs. **Mitigation:** The results of RQ3 show that variations happen mostly within patterns that have the lowest number of samples. All analyses were done within the top 45k patterns out of 328k patterns with at least 3 occurrences to increase confidence.

The pattern interpretation was supported by the content track but was ultimately still done manually, which leaves open the possibility for error. **Mitigation:** The interpretation was done by two researchers separately, discussing the results until a conclusion was reached.

### External validity

Further, the project selection might not be representative. All analyzed code was written in Java and came from highly-rated open-source projects. Therefore, the results might not generalize to other languages. The patterns might also differ for proprietary projects. **Mitigation:** The dataset is a large sample of 1000 projects. It covers a diverse range of project sizes and different numbers of contributors, with over 55k distinct contributors combined.

## Related work

### Source code diffing

GumTree, proposed by Falleri et al.^23^, generates fine-grained source code diffs in a multi-phase process consisting of a top-down phase and a bottom-up phase. The top-down phase maps the nodes of the AST before any changes to the nodes of the AST after all changes, employing a heuristic. Then, in the bottom-up phase, an edit script consisting of *add*, *delete*, *modify*, and *move* actions is generated.

Several adaptations of GumTree have been proposed. Dotzler et al.^32^ present five optimizations applicable to GumTree as well as other change distillers, in an effort to shorten edit scripts by increasing the use of *move* operations. Huang et al.^33^ propose an approach that compresses edit scripts to a coarser granularity. After running GumTree to extract fine-grained code changes, the changes are grouped into a more human-understandable high-level representation.

ChangeDistiller^34^ is an AST-based diffing algorithm based on an algorithm by Chawathe et al.^35^ that extracts changes from general hierarchically structured data. A key idea is to use the Chawathe algorithm for inner nodes while using bigram string similarity to match code statements on the ASTs’ leaves.

Alexandru et al.^36^ propose Lean Language-Independent Software Analyzer (LISA), a genetic framework to represent and analyze source code repositories. Using LISA, the whole project with all revisions can be represented as a single graph. A relevant application is HyperDiff^37^, which is based on a similar approach similar to LISA called HyperAST^38^. The idea of HyperDiff is to improve GumTree by reusing intermediate computation steps, both of the initial code parsing and Gumtree’s diffing steps. All discussed approaches focus on fine-grained edits and inspect changes within a single file at a time. Therefore, they do not detect changes that move between files, or do overarching operations.

### Graph-based pattern mining

Nguyen et al.^17^ present the pattern mining approach CPatMiner for concrete graph-based patterns. It differentiates node types for operations, data, and control flow. Using a heuristic pattern mining method, frequent patterns, and their constant context are mined. The method is evaluated on 332*k* graphs consisting of 8*M* nodes, mining a total of 25*K* patterns. They found that $7\%$ of patterns are cross-project while covering $48.5\%$ of all code changes. They analyze the commonality of the patterns they mined within and across developer teams. $43\%$ of developers shared all their patterns with other developers, while $31\%$ of projects shared all their patterns with other projects. Even though the patterns presented by Nguyen et al. are on a lower abstraction than the ones presented by us, they paint a consistent picture. They argue that patterns are commonly shared between contributors and within projects, and reused over time. Our study confirms that there are these types of shared patterns. However, we could also show that there is a diverse set of patterns that represents the opposite: mostly personal or project-specific patterns.

Dilhara et al.^39^ propose JavaFyPy, an approach to transform Python ASTs into Java ASTs. The approach is based on the Java-specific CPatMiner^17^ that was made applicable for Python through this conversion. They additionally denoised the results of CPatMiner using RefactoringMiner 2.0^40^. The approach is evaluated with a study on fine-grained change patterns using a corpus of 1000 machine learning Python projects. They were able to extract four major trends: (1) transformation to context managers, (2) conversion of for-loops into domain-specific abstraction, (3) update of API usage, and (4) use of advanced language features.

These patterns are by construction low-level, and specific to Python and the machine learning domain. The individual patterns are typically concerned with the usage of APIs such as NumPy, and the usage of Python-specific features such as with or efficient short forms such as {} for dictionary constructions. These do not have a direct counterpart in Java. However, they confirm that patterns can be *frequent* in many different ways, and the results are strongly based on the underlying dataset. The conglomeration of patterns into more abstracted trends also supports the idea of more general patterns with a high number of instances. One such general pattern is trend (1) with 1237 instances, where the content track could be used to oversee and categorize them.

Higo et al.^41^ propose TC2P, advancing the frequent multiset-based mining technique by Negara et al.^19^. They use GumTree^23^ in order to extract fine-grained edit scripts and extract a tree from their AST relations. Since the complexity of the frequent tree mining problem is much lower than that of the frequent subgraph mining problem, much more efficient algorithms can be employed, such as FREQT^42^. In contrast to CPatMiner, TC2P was able to detect code moves using the parent node’s information, while CPatMiner could capture statement-level replacements using their representation of control and data dependencies. Compared to our method, TC2P works on the abstraction level of files. They, therefore, do not connect file-overarching changes. TC2P considers lower-level changes to individual statements, while our method aims for high-level transformations and finds additional connections based on the tokens’ names.

Janke et al.^18^ present a mining method for abstract patterns with semantic connections of code tokens. The mining uses the exact frequent subgraph mining algorithm gSpan. The method is evaluated on 115*k* graphs with 3*M* nodes. In a small study of 7 projects, they found that patterns are 2.7 times more likely to be found in the project they were mined from compared to previously unseen projects.

### Refactorings and code maintenance

Santos et al.^16^ used a manual process to extract complex project-specific transformation patterns. Their results show that often, application opportunities for such patterns are missed. They present a total of 6 patterns with 6 to 66 instances. Similar to our method, they focused on high-level patterns on the level of classes, interfaces, methods, and attributes. The patterns cover similar themes to ours, with high-level refactorings and the delegation of functionality playing a large role. However, they did not abstract from individual names and therefore, patterns are not directly comparable. Despite that, there is an overlap in the patterns. The pattern pIII they found describes how an interface is added and consequently, a method previously modeling that functionality is removed, which is complementary to our pattern P12121 which describes how an interface is removed and the functionality is replaced by a method. They conclude that automated support for transformation patterns could be beneficial.

Silva et al.^43^ analyzed a set of 124 Java projects to identify motivations for common refactoring types, such as extracting a method to “make reusable code accessible to other methods” or “introduce an alternative method signature” or moving a class to “put it in a conceptually more relevant package”. Their work shows that there are many different motivations, not only between different refactoring types but also for the same kinds of refactorings. They also found that the IDE plays an important role in supporting refactorings.

Gharehyazie et al.^44^ selected 5, 753 Java projects on GitHub and analyzed them for code clones. They found that cross-project code reuse is common, with up to $5\%$ of code being cross-project clones. Their results suggest cloning is more common among experienced and active developers. To build the pattern database, they advise using similar projects, based on different criteria such as them being in the same domain, developed by the same organization, the same developers, or even the same software. These selection criteria are similar to the dimensions we analyzed in order to categorize patterns.

Higo et al.^45^ propose a system to use cross-project patterns to make change suggestions. The approach is based on a pattern database that needs to be populated with a diverse set of change patterns. They analyzed 325*k* commits of 122 projects from the Apache software foundation.

Nguyen et al.^6^ present a large study for code repetitiveness of 2.5*BN* changed lines of code. They come to the conclusion that $70\%$ to $100\%$ of small changes are repetitive, noticing an exponential decline as the changes get larger. They note a higher repetitiveness of code within a project, which is consistent with our finding that projects have a set of specific patterns. Tsantalis et al.^46^ propose a methodology to automatically identify opportunities to improve code quality by moving a method between classes. For this, they define a distance metric between attributes/methods, as well as a set of quantifiable rules to detect feature envy. In a similar manner, Tsantalis et al.^47^ detected opportunities for the “extract method” refactoring. It shows a possible application of refactoring patterns, integrating it into the Eclipse Plugin JDoedorant.

Trautsch et al.^48^ analyze commits from an angle of developer intent which they judged using commit messages. They categorized changes into corrective changes and perfective changes, finding that corrective changes are larger and more complex. Perfective changes could be observed to be more common in smaller and less complex files. Extracting different types of intent behind a change as done by Wang et al.^49^ could be incorporated into our approach as additional characteristics that help to interpret and query patterns. We could already show that different patterns correlate with different sizes and timing of commits, which is a hint that these patterns might also correlate with different intentions.

A different approach to analyzing source code is by the use of machine learning models. Wang et al.^50^ encode a program’s AST into a token vector to automatically learn a representation. They use this to predict bugs in previously unseen source code. A common difficulty of machine learning approaches is the explainability of results, which carries over to just-in-time bug prediction, as investigated by Aleithan^51^.

## Conclusion and future work

We mined 45, 000 high-level code change patterns from a large set of 1000 open source Java projects. We presented a categorization into different pattern kinds based on 7 dimensions: project, contributors, edit type, object relations, timing, edit context, and pattern frequency. We presented 13 patterns that showcase the large diversity between patterns based on these external factors.

Our results show that code change patterns are diverse and cannot easily be seen as a single monolithic entity. They are highly dependent on their usage context. Our study shows that there are patterns commonly shared between developers and across the borders of projects. It also showed that there is an important counterpart to this in patterns that only exist under specific circumstances. Examples are project-wide coding conventions (explicitly enforced or implicitly transferred between peers), specific ways of interacting with an API, and even personal programming styles.

Overall, this points towards the possibility of improving pattern analysis and recommendation by valuing patterns that fit the context, rather than ones that are generally frequent. In future work, results could be extended towards other languages. Also, pattern recommendation techniques could be combined with context-specific knowledge stemming from the proposed dimensions.

## Data Availability

The datasets generated during and/or analysed during the current study are available in the dataverse repository at https://dataverse.harvard.edu/privateurl.xhtml?token=ad7488fa-6f47-4d36-866b-9b0a26a1077b.
